# Extractive adsorption of 1,3-propanediol on a novel polystyrene macroporous resin enclosing medium and long-chain alcohols as extractant

**DOI:** 10.1186/s40643-023-00646-3

**Published:** 2023-04-20

**Authors:** Wen-Bo Sui, Lu-Sheng Huang, Xiao-Li Wang, Xu Zhou, Ya-Qin Sun, Zhi-Long Xiu

**Affiliations:** School of Bioengineering, Liaoning, Dalian University of Technology, No.2 Linggong Road, Ganjingzi District, Dalian, People’s Republic of China 116024

**Keywords:** Extractive adsorption, Resin synthesis, Characterization, 1,3-Propanediol, Sorbent concentration effect

## Abstract

Extractive adsorption is an integrated separation method employing a novel resin with both particle and liquid characteristics in terms of adsorption and extraction. In this study, the novel extractive adsorption polystyrene-divinylbenzene (PS-DVB) macroporous resin was synthesized by suspension polymerization, in which *n*-octanol (OL-PS-DVB) or mixed alcohols of n-octanol, undecyl alcohol, and tetradecyl alcohol (MA-PS-DVB) were added as porogen and enclosed in the resin skeleton after the reaction. The characterization of the two novel resins of OL-PS-DVB and MA-PS-DVB showed that they have large specific surface areas of 48.7 and 17.4 m^2^/g, respectively. Additionally, the two synthesized resins have much higher static adsorption capacities of 1,3-propanediol (511 and 473 mg/g) and dynamic adsorption capacities (312 and 267 mg/g) than traditional resins, because extractants enclosed in the resin can increase the adsorption capacity. Through Langmuir equation, the theoretical static maximum adsorption capacity of the mixed alcohols resin is 515 mg/g at 298 K and Gibbs free energy change of adsorption was -3781 J/mol, indicating that the adsorption process was spontaneous. In addition, the sorbent concentration effect in the resin was generated at high 1,3-propanediol (1,3-PDO) concentrations. The fitting of the Flocculation model can reveal that there is a possible relation between adsorption and flocculation. Compared to OL-PS-DVB, MA-PS-DVB showed better performance in the recovery yield of 1,3-PDO and other byproducts, the removal rates of the inorganic salt and protein, and the efficiency of recycled resin. For MA-PS-DVB, the recovery of 1,3-PDO, butyrate acid, acetic acid, and residual glycerol was 97.1%, 94.7%, 93.3%, and 90.3%, respectively. Simultaneously, the resin of MA-PS-DVB could remove 93.8% of inorganic salts and 90.9% of proteins in the concentrated fermentation broth. The two synthesized resins of OL-PS-DVB and MA-PS-DVB still had 90% or 92% of capacity for extractive adsorption of 1,3-propanediol after 10 times of recycling, which exhibited potential application in the separation of 1,3-propanediol from fermentation broth.

## Highlights

- The novel extractive adsorption PS-DVB macroporous resin was synthesized
- The structure of the synthesized resins was characterized
- The static and dynamic adsorption behavior of synthesized resins was investigated
- The sorbent concentration effect was generated at high 1,3-PDO concentrations
- Synthesized resins showed excellent performance in separation 1,3-PDO from broth

**Graphical Abstract:**

## Graphical abstract

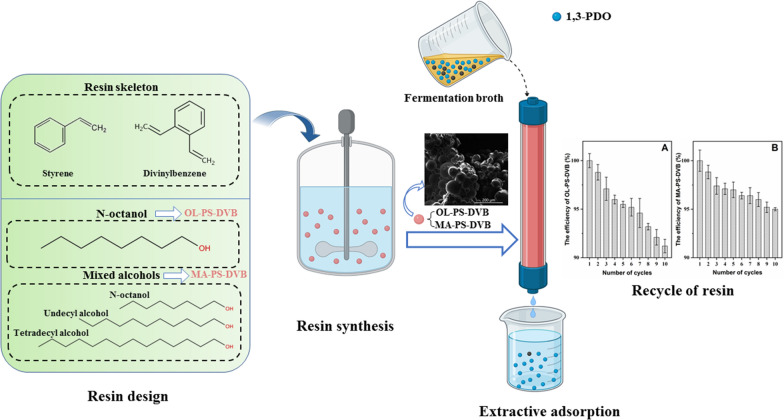

## Introduction

Recently biofuels instead of fossil fuels have been paid attractive attention worldwide due to serious environmental and climate damage caused by the extensive use of fossil fuels (Kilbane 2016; Zweig 2021). The major biofuels are currently bioethanol and biodiesel, of which glycerol is their byproduct. The utilization of a large amount of glycerol to produce products with high-added value is a great challenge. Many attempts have been tried, in which bioconversion of glycerol to 1,3-propanediol is one of the valuable ways. 1,3-Propanediol (1,3-PDO) is a valuable bulk chemical that has been widely applied in many fields (Wehner et al. 2007; Gohil 2010). For example, 1,3-PDO has been used as a moisturizer and antibacterial agent for cosmetics due to its good moisturizing effect and low heterogeneity (Liu et al. 2021). For 1,3-PDO, the separation process plays an important role in its microbial production and the cost of downstream processing makes a very high portion of the total production cost, mounting up to about 50–70% (Sun et al. 2018). The separation of 1,3-PDO from the fermentation broths becomes a bottleneck for its industrialization.

The separation of 1,3-PDO from the fermentation broths is a complex task because the fermentation broth is a mixed system, including cells, biomacromolecules (nucleic acids, polysaccharides, and proteins), residual substrates, salts (organic salts formed during fermentation and residual inorganic salts in medium), pigments, and other byproducts (butyrate, 2,3-butanediol, lactate, acetate, succinate, etc.) (Xiu and Zeng 2008; Johnson and Rehmann 2016; Hao et al. 2006). The general separation procedure for bio-chemicals is to first remove cells and biomacromolecules. Then, the second step is the removal of impurities and the primary separation of 1,3-PDO. A variety of separation methods have been investigated to remove cells, biomacromolecules, or/and salts, such as ultrafiltration (Li et al. 2001a, 2001b), nanofiltration (Bastrzyk and Gryta 2015), alcohol precipitation (Zhang et al. 2006), electrodialysis (Wu et al. 2011; Hao and Liu 2005), or ion exchange desalination (Liu et al. 2020). These technologies are difficult to apply due to high operating costs and large product loss (Fokum et al. 2021). The extraction method is efficient for the separation of target products and has been widely used in many areas, including the separation of 1,3-PDO (Sun et al. 2018; Boonsongsawat et al. 2010). However, it is difficult for traditional liquid–liquid extraction using organic solvents such as ethyl acetate and soybean-derived biodiesel as extractants to separate 1,3-PDO effectively from the fermentation broth (Boonsongsawat et al. 2010; Adhami et al. 2009). Multi-stage extraction and a large amount of extractants are required to increase extraction recovery. Moreover, reactive extraction by aldehyde or isobutyraldehyde, and salting-out extraction were also developed to separate 1,3-PDO (Alejski et al. 2017; Adams and Seider 2009; Li et al. 2019, 2009; Wu and Wang 2012). Although these methods improve extraction performance obviously, some problems are still needed to solve for their industrial application. For example, aldehyde extractants used in reactive extraction are easy to be oxidized into corresponding carboxylic acids in long-term circulation. A large amount of salt for salting-out extraction needs to be recycled (Zhang et al. 2021).

Chromatography is a widely used separation method with high selectivity under mild conditions. Its application in 1,3-PDO separation mainly focuses on ion-exchange chromatography. Different types of ion-exchange resins were currently used to desalt the 1,3-PDO fermentation broth (Liu et al. 2020). Moreover, sulfonated polystyrene cationic resin and basic acrylic resin were also applied to adsorb 1,3-PDO from the fermentation broth (Hilaly and Binder 2002; Roturier et al. 2000). The adsorption capacity of sodium or calcium polystyrene sulfonate resin used for separating 1,3-PDO was about 5.4 mg/mL and 25.5 mg/mL, respectively, while 0.6 mg/mL was for basic acrylic resin. These resins have too low an adsorption capacity of 1,3-propanediol to be applied on an industrial scale.

Due to the advantages of extraction and absorption, extractive chromatography integrating extraction and chromatography into a unit operation might be a good choice for 1,3-PDO separation (Zakhodyaeva and Voshkin 2013; Hyotylainen and Riekkola 2004). In this study, novel extractive adsorption resins were synthesized by suspension copolymerization for the separation of 1,3-PDO from fermentation broth. The synthesized polystyrene-divinylbenzene (PS-DVB) macroporous resins were characterized to determine hydroxyl group content, adsorption isotherms, and pore size of resin, as well as the adsorption capacity through static and dynamic adsorption. The adsorption equilibrium data were simulated and analyzed according to Langmuir isotherm model and Freundlich isotherm model. In addition, the sorbent concentration effect was described according to the Flocculation model. Moreover, the breakthrough curve was simulated according to Yoon–Nelson model and Thomas model to predict the absorption behavior.

## Materials and methods

### Materials

1,3-PDO standard was purchased from Shanghai Adamas-beta Technology Co., Ltd. Acetic acid (HAc) and butyric acid (BA) standards were purchased from Sinopharm Chemical Reagent Co., Ltd. All other chemicals were of analytical grade. The fermentation broth was produced using fed-batch fermentation by *Clostridium butyricum* DL07 (Wang et al. 2020). The fermentation broth was filtered by cellulose triacetate hollow fiber membrane with a cut-off molecular weight of 5000 Dalton to remove almost all bacterial cells and most proteins. And the obtained filtrate was then concentrated in two folds by a rotary evaporator. The concentrated broth was used in the extractive adsorption of 1,3-PDO. The concentration of 1,3-PDO, BA, HAc, and residual glycerol in the concentrated broth was 174.5 g/L, 19.4 g/L, 16.1 g/L, and 11.4 g/L, respectively.

### Synthesis of extractive adsorption resins

The resins were synthesized by suspension copolymerization. The organic phase was formed by mixing styrene (15 g), porogen (15 g), and divinylbenzene (10 g) at a mass ratio of 1.5:1.5:1 in an Erlenmeyer flask. Porogen was n-octane, n-octanol, or mixed alcohols (n-octanol, undecyl alcohol, and tetradecyl alcohol at a molar ratio of 15:4:6). Additionally, 0.5 g azobisisobutyronitrile (AIBN) was added into the organic phase. Then, 0.1 g sodium dodecyl sulfonate, 0.5 g calcium carbonate, and 5 g gelatin were dissolved in distilled water to form the water phase (500 mL). The water phase and organic phase were mixed in a reactor for 20 min at 45 °C and heated up to 85 oC at a stirring speed of 200 r/min for 6 h. Finally, the mixed system was heated up to 90 °C and reacted for 30 min. The obtained product was washed three times with distilled water and the resins in 10–200 mesh were selected. The synthesized resin using n-octane as porogen (OE-PS-DVB) was used as a blank control. The resins using n-octanol and mixed alcohols as porogen were named OL-PS-DVB and MA-PS-DVB, respectively. Their pore size distribution, the content of the hydroxyl group, and extractive adsorption capacity would be characterized.

### Static adsorption experiment

The resins of OE-PS-DVB, OL-PS-DVB, and MA-PS-DVB (1.0 g) were added into the test tube with the concentrated 1,3-PDO fermentation broth at 25 °C, standing for 24 h after mixing. The concentration of 1,3-PDO was measured by HPLC before adding resin and after standing. The static adsorption capacity of the resin was calculated by Eq. (1).

1 $$Q=\frac{({c}_{0}-{c}_{e})V}{m}$$

where *c*_*0*_ and *c*_*e*_ are the concentrations of 1,3-PDO in the aqueous solution (mg/L) at the initial and equilibrium stage, respectively. *V* is the volume of the solution (L) and *m* is the weight of the resin (g), *Q* is the adsorption capacity for 1,3-PDO (mg/g).

### Determination of adsorption isotherm

The resin of MA-PS-DVB (0.5 g) was added to the standard solutions of 1,3-PDO at different concentrations and temperatures, respectively. The concentrations of 1,3-PDO standard solutions are 25, 50, 75, 100, 150, and 200 g/L, respectively. The temperature is 298, 308, and 318 K, respectively. The concentration of 1,3-PDO at equilibrium was measured by HPLC after standing for 24 h and the data were fitted according to Langmuir isotherm model and Freundlich isotherm model (Juang and Shiau 1999). The Langmuir isotherm model can be expressed as Eq. (2) shown.

2 $$Q=\frac{k{{c}_{e}Q}_{m}}{1+k{c}_{e}}$$

where *k* is Langmuir equilibrium constant, *Q*_*m*_ is the saturated adsorption capacity of resin (mg/g) and *c*_*e*_ is the concentration of 1,3-PDO at equilibrium (g/L).

Gibbs energy change in the adsorption process is calculated according to Eq. (3).

3 $${\Delta G}^{\mathrm{o}} =-\mathrm{RTln}K$$

where *K* = *Q*_*m*_*/Q*, *R* is the gas constant (8.314 J/(mol·K)), and *T* is the temperature (K).

The Freundlich isotherm model can be expressed as Eq. (4) shown.

4 $$Q={k}_{F}{c}_{e}^{1/n}$$

where *k*_*F*_ and *n* are the adsorption constants.

### Determination of sorbent concentration effect

The resins of OL-PS-DVB and MA-PS-DVB (0.5 g) were added into 5 mL standard solutions of 1,3-PDO at different concentrations and temperatures, respectively. The concentration of 1,3-PDO standard solutions is 200, 300, 400, 500, 600, 700, 800, and 900 g/L, respectively. The temperature is 298, 308, and 318 K, respectively. The concentration of 1,3-PDO at equilibrium was measured by HPLC after standing for 24 h and the data were fitted according to the Flocculation model (Helmy et al. 2000). The model can be expressed as Eq. (5) shown.

5 $${X}_{m}^{1/2}={a}_{0}+{a}_{1}{c}_{e}$$

where *X*_*m*_ = *Q* × *c*_*e*_, *a*_*0*_ and *a*_*1*_ are the constants.

### Determination of breakthrough curve

The resins of OL-PS-DVB and MA-PS-DVB (10 g) were added into a column (Φ1.5 × 19 cm) by the wet packing method. The breakthrough curve was determined by pumping concentrated 1,3-PDO fermentation broth into the column at a flow rate of 1 mL/min and 25 °C. The effluents from the column were collected and the concentration of 1,3-PDO in the effluents was measured by HPLC. The breakthrough curve was fitted by Thomas and Yoon–Nelson model (Ipek et al. 2013). The total dynamic adsorption of 1,3-PDO was calculated as Eq. (6) shown.

6 $$Q=\frac{F}{m}\cdot {c}_{0}\int_{0}^{t}\left(1-\frac{{c}_{e}}{{c}_{0}}\right)\mathrm{d}t$$

where *F* is the flow rate (mL/min), *m* is the weight of the resin (g), *c*_*e*_ is the concentration of 1,3-PDO (mg/mL) during adsorption at the time of *t* (min), *c*_*0*_ is the initial concentration of 1,3-PDO (mg/mL).

The Thomas model can be expressed as Eq. (7) shown.

7 $$\frac{{c}_{e}}{{c}_{0}}=\frac{1}{1+\mathrm{exp}[{K}_{T}\left({q}_{0}m-{c}_{0}{V}_{eff}\right)/{Q}_{v}]}$$

where *k*_*T*_ is Thomas rate constant (mL/(min·mg)), *q*_*0*_ is the equilibrium uptake of 1,3-PDO (mg/g), *m* is the quality of resin in the column (g), *Q*_*v*_ is the volumetric flow rate (mL/min), and *V*_*eff*_ is the volume of effluents (mL).

The Yoon–Nelson model can be expressed as Eq. (8) shown.

8 $$\frac{{c}_{e}}{{c}_{0}}=\frac{1}{exp({k}_{YN}\tau -{k}_{YN}t)+1}$$

where *k*_*YN*_ is the rate constant (min^−1^), and *τ* is the time required for a 50% breakthrough of 1,3-PDO (min).

### Recycle of resins

After the resins of OL-PS-DVB and MA-PS-DVB were completely adsorbed in the column (Φ1.5 × 19 cm), 1,3-PDO was eluted with water. And the concentration of 1,3-PDO in the elute was measured by HPLC. A similar operation was repeated 10 times to determine the recycling ability of resins. The efficiency of resin (*E*) was calculated according to Eq. (9).

9 $$E=\frac{{Q}_{i}}{{Q}_{1}} i=1,\dots ,n$$

where *Q*_*1*_ is the adsorption capacity of resin for the first time (mg/g), *Qi* is the absorption capacity of resin for each absorption (mg/g), and *n* is the number of absorption cycles.

### Analytical methods

The concentrations of 1,3-PDO, HAc, BA, and glycerol in the samples were determined by HPLC as described previously (Dai et al. 2017). The protein concentration was determined by Bicinchoninic acid (BCA) method and the inorganic salt content was measured by a conductivity meter (Smith et al. 1985).

The pore size distribution in resins was calculated by the BET equation which was obtained by a physicochemical adsorption instrument (Wu et al. 2003). The hydroxyl group in resins was measured by Fourier Transform Infrared Spectrum (FTIR). The hydroxyl content was determined according to the hydroxyl acetylation reaction (He 2015). Under the catalysis of pyridine, alcohol reacts quantitatively with acetic anhydride at 90–100 °C. The content of alcohol was calculated by acid–base titration using the macroporous resins as blank control. The operations were as follows: First, the acetylation reagent was obtained by mixing 10 mL acetic anhydride with 90 mL pyridine. Then, the three resins were weighed accurately to be 0.4 g. The resins and 20 mL acetylation reagent were added into a distillation flask with a straight condenser tube. The reaction solution was heated to 95 °C. After back-flowing for 1 h, 20 mL distilled water and 2–3 drops of phenolphthalein indicator (10 g/L) were added. The product (acetic acid) was titrated using a 1 mol/L NaOH standard solution. The volume of NaOH standard solution consumed was recorded at the titration endpoint. The calculation formula is as follows:

10 $$W=\frac{({V}_{1}-{V}_{2})CM}{m}\times 100\%$$

where *V*_*1*_ is the consumption of NaOH standard solution in the blank control (L), *V*_*2*_ is the consumption of NaOH standard solution in the experiment (L), *C* is the concentration of NaOH (mol/L), *m* is the mass of resin (g), *M* is the molar mass of the hydroxyl group (17.01 g/mol).

### Statistical analysis

The experiments were repeated three times and the value stood for the average. The differences in mean values were evaluated using the analysis of variance (ANOVA) method, and standard deviations were calculated to verify the reliability of the results.

Langmuir model, Freundlich model, Flocculation model, Thomas model, and Yoon–Nelson model are fitted by the least-squares method. The model fitting of experimental data was analyzed using Origin software Version 9.4. The regression coefficient (R^2^) from the analysis of variance (ANOVA) was used to check the statistical significance (p ≤ 0.05).

## Results and discussion

### Screening of extractants and porogens

It is key for extractive adsorption to choose a suitable solvent used as both extractant and porogen. Medium and long-chain fatty alcohols can be dissolved in the organic phase to act as porogenic agent due to their hydrophobicity. At the same time, they also have a certain extraction capacity for 1,3-PDO. Generally speaking, the longer the fatty chain of alcohols is, the more hydrophobic and the less extraction capacity. The recovery of 1,3-PDO is 23.2%, 12.2%, and 10.8% using n-octanol, undecyl alcohol, and isotridecanol as extractants, respectively (Sui et al. 2022). Moreover, mixed alcohols with medium and long-chain can not only increase hydrophobicity higher than medium-chain alcohol but also increase recovery of 1,3-PDO more than long-chain alcohol. In addition, mixed alcohols of n-octanol, undecyl alcohol, and tetradecyl alcohol achieved a 1,3-PDO extraction yield of 16.8% at 25 °C at a molar ratio of 15:4:6 (Sui et al. 2022). Therefore, in this study, n-octanol and mixed alcohols (n-octanol, undecyl alcohol, and tetradecyl alcohol in a molar ratio of 15:4:6) were selected as extractants, but also as porogens, respectively.

The resin skeleton was obtained through suspension copolymerization of styrene and divinylbenzene. During the reaction, n-octanol, mixed alcohols, and n-octane were dispersed in microdroplets and acted as pore-making agents. As a result, three resins named OE-PS-DVB (n-octane as porogen), OL-PS-DVB (n-octanol as porogen), and MA-PS-DVB (mixed alcohols of n-octanol, undecyl alcohol, and tetradecyl alcohol as porogen) were synthesized and will be characterized.

### Characterization of PS-DVB macroporous resins

**Fig. 1:**
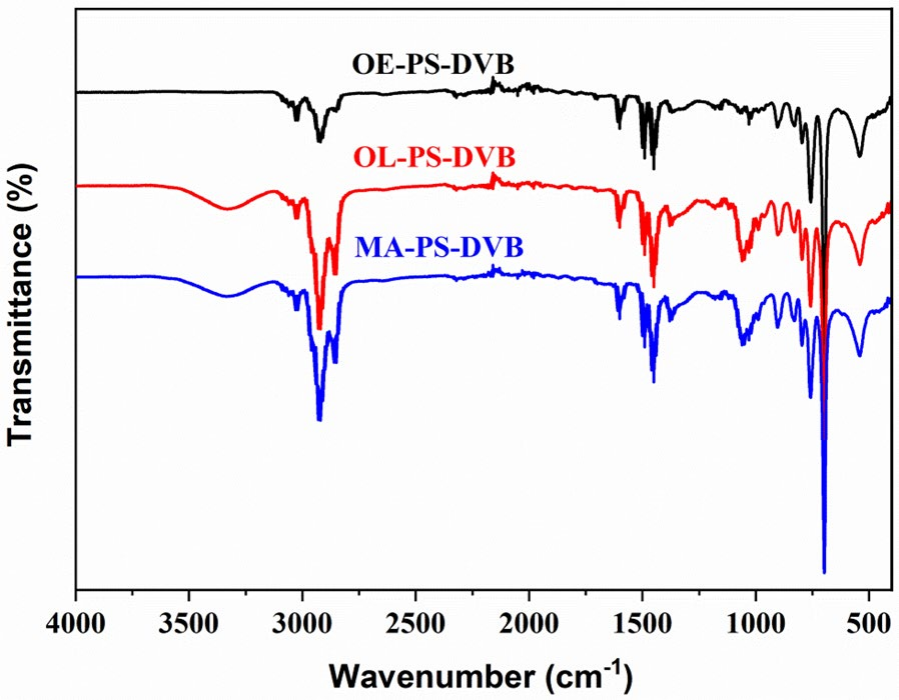
FTIR spectra of macroporous resins of OE-PS-DVB, OL-PS-DVB, and MA-PS-DVB

The structure of resins was characterized by Fourier Transform Infrared Spectrum (FTIR), hydroxyl content, and pore size distribution. The infrared spectra of three resins are shown in Fig. 1. According to the previous reports about the skeleton structure of resins, the peaks at 700 cm^−1^, 750 cm^−1^, 1460 cm^−1^, and 2975–2800 cm^−1^ represent the skeleton deformation vibrating ring, C-H plane vibration δ ring of the benzene ring, CH_2_ shear deformation vibration, and alkane CH bond, respectively (Lutz and Vandermaas 1988; Sultanov 1984; Miyazaki and Fujii 2017). A comparison of the alkane CH bond range among the three resins showed that the absorption peaks of OL-PS-DVB and MA-PS-DVB are larger than OE-PS-DVB because OL-PS-DVB and MA-PS-DVB contain more CH_2_ structure in the linear alcohols. Different from OE-PS-DVB, OL-PS-DVB, and MA-PS-DVB have absorption peaks at 3400–3300 cm^−1^, representing the alcoholic hydroxyl groups in porogens. This indicated that the alcoholic porogens were successfully enveloped in the synthesized resins. Using hydroxyl acetylation reaction and according to Eq. (10), the hydroxyl content was determined to be 7.4% and 6.1% in OL-PS-DVB and MA-PS-DVB, respectively. Correspondingly, n-octanol and mixed alcohols in OL-PS-DVB and MA-PS-DVB were 56.3% and 56.7%, respectively.

**Fig. 2:**
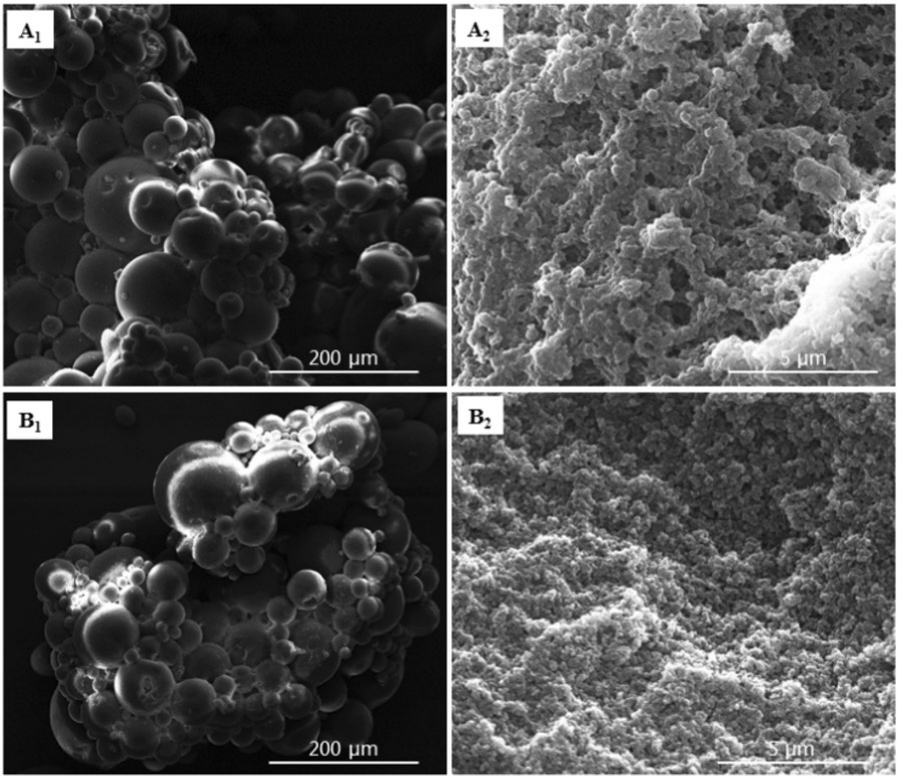
SEM images of the synthesized resins of OL-PS-DVB (**A**) and MA-PS-DVB (**B**)

The synthesized resins of OL-PS-DVB and MA-PS-DVB were comprehensively observed using a scanning electron microscope (SEM) and the results indicated that the two resins have a similar structure (Fig. 2). The synthesized resins were both spherical and had macroporous structure.

**Fig. 3:**
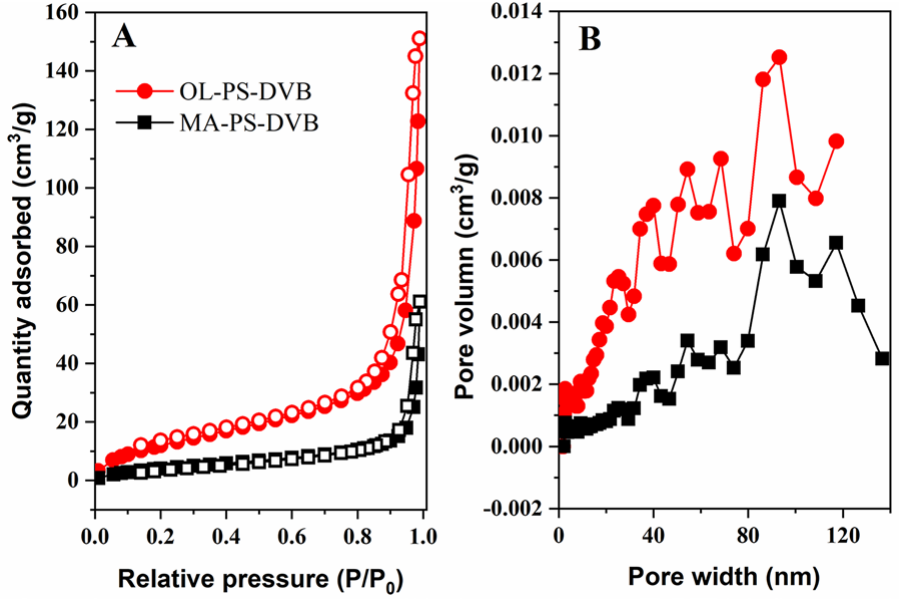
Adsorption (filled) and desorption (empty) isotherms of N_2_ at 77 K for synthesized OL-PS-DVB and MA-PS-DVB (**A**); Pore size distribution curves of OL-PS-DVB and MA-PS-DVB (**B**)

The adsorption–desorption of nitrogen for OL-PS-DVB and MA-PS-DVB were investigated and the results are shown in Fig. 3A. The pore structure and size distribution of OL-PS-DVB and MA-PS-DVB (Fig. 3B) were determined using BET model fitting according to adsorption–desorption of nitrogen. The hysteresis loop of adsorption isotherm without a saturated adsorption platform under high pressure illustrated the existence of irregular pore structure in resins. The pore structure was mainly a slit or wedge structure according to the hysteresis loop type (Sing 1998; Yuan et al. 2011). The specific surface areas of OL-PS-DVB and MA-PS-DVB were 48.7 and 17.4 m^2^/g, respectively, according to the BET model (Wu et al. 2003). The pore size distribution indicated that both OL-PS-DVB and MA-PS-DVB had microporous, mesoporous, and macroporous structures in a wide range of pore size distribution with an average pore diameter of 19.2 and 21.7 nm for OL-PS-DVB and MA-PS-DVB, respectively. Compared with OL-PS-DVB, MA-PS-DVB had a more macroporous structure.

### Static extractive adsorption

The static adsorption capacity of three resins for the concentrated 1,3-PDO broth illustrated that OL-PS-DVB and MA-PS-DVB performed better than the control resin (OE-PS-DVB), being 511 and 473 mg/g vs. 102 mg/g, i.e., 5 and 4.6 times, respectively. These results showed that the synthesized PS-DVB macroporous resin had some adsorption capacity for 1,3-PDO, and the immobilization of extractant in the resins could greatly improve the adsorption capacity. This novel kind of resin exhibited both extraction and adsorption characterization, also called extractive adsorption resin. The adsorption capacity of OL-PS-DVB, MA-PS-DVB, and OE-PS-DVB also indicated that the adsorption effect of resin skeleton took about 20% of total capacity, and the extraction effect of medium and long-chain alcohols was about 80%.

Compared with the adsorption capacity of traditional ion-exchange resin, e.g., 297 mg/g, the extractive adsorption resins have a great advantage due to the combination of adsorption and extraction (Wang et al. 2014). On the other hand, extractive adsorption resins also have higher extraction efficiency than traditional liquid–liquid extraction. For the 1,3-PDO fermentation broth, only 26 mg and 20 mg 1,3-PDO could be extracted into the organic phase by 1 g n-octanol and mixed alcohols (n-octanol, undecyl alcohol, and tetradecyl alcohol in a molar ratio of 15:4:6) as extractant, respectively (Sui et al. 2022). For extractive absorption resin, at least 473 mg 1,3-PDO was extracted using 1 g synthesized OL-PS-DVB and MA-PS-DVB. If only considering extraction, more than 25 times 1,3-PDO were extracted into the same amount of extractants enveloped in extractive adsorption resin as traditional extraction. Small particles and irregular pore structures of extractive adsorption resin could provide large specific surface areas inside and exterior resin, resulting in much contact chances between the target and extractants. Each particle of resin is equivalent to a small extractor, and the whole adsorption process is similar to multi-stage continuous extraction, leading to a great improvement in extraction efficiency in extractive adsorption resins. Table 1 summarizes the studies on the recovery of 1,3-PDO by ion exchange resins. The adsorption capacity of OL-PS-DVB and MA-PS-DVB resin on 1,3-PDO is among the higher values reported in the literature.

**Table 1:** Adsorption studies using ion-exchange resin for 1,3-PDO recovery

Resin	Matrix	Adsorption (mg/g resin)	References
D201	Polystyrene-divinylbenzene	146	Liu et al. (2020)
D315	Acrylic	113	Liu et al. (2020)
001 × 7 H-form	–	297	Wang et al. (2014)
PS-3NB	Polystyrene-divinylbenzene	18	Zheng et al. (2020)
PS-SBT	Polystyrene-divinylbenzene	17	Zheng et al. (2020)
UBK555 Na-form	Polystyrene sulfonate	5.4 mg/mL	Hilaly and Binder 2002)
UBK555 Ca-form	Polystyrene sulfonate	25.5 mg/mL	Hilaly and Binder (2002)
OL-PS-DVB	Polystyrene-divinylbenzene	511	This work
MA-PS-DVB	Polystyrene-divinylbenzene	473	This work

### Effect of temperature on extractive adsorption

Increasing temperature is conducive to the extractive adsorption process and the raise is more influenced by the nature of extraction rather than adsorption. It is important to establish the adsorption isotherm, which can be used to predict the adsorption parameters reliably, compare the adsorption performance in 1,3-PDO standard solution at different temperatures and optimize the design of an adsorption system (Yuan et al. 2020). The adsorption isotherm of MA-PS-DVB was investigated and calculated. The adsorption isotherm of MA-PS-DVB fitted well with the Langmuir model and Freundlich model and the fitting equation is shown in Tables 2 and 3. Both two models have a good fitting effect in which the determination coefficient R^2^ can reach more than 0.975 at investigated temperatures. Calculated by the Langmuir equation, the theoretical static maximum adsorption capacities of MA-PS-DVB are 516, 595, and 629 mg/g at 298, 308, and 318 K, respectively. In the above temperatures, Gibbs free energy changes of adsorption are -3781, -4018, and -4169 J/mol, respectively, indicating that the adsorption process was spontaneous.

**Table 2:** Langmuir model fitting results of MA-PS-DVB

Temperature (K)	Equation	R^2^	△G^o^ (J/mol)*	Q_m_ (mg/g)
298	c_e_/Q = 0.00194c_e_ + 0.19369	0.984	− 3781	516
308	c_e_/Q = 0.00168c_e_ + 0.18178	0.999	− 4018	595
318	c_e_/Q = 0.00159c_e_ + 0.16619	0.992	− 4169	629

^*^△G^o^ calculated using data from 1,3-PDO standard solution at 25 g/L

**Table 3:** Freundlich model fitting results of MA-PS-DVB

Temperature (K)	Equation	R^2^
298	logQ = 0.559logc_e_ + 1.273	0.992
308	logQ = 0.5721logc_e_ + 1.292	0.994
318	logQ = 0.5541logc_e_ + 1.36	0.995

**Fig. 4:**
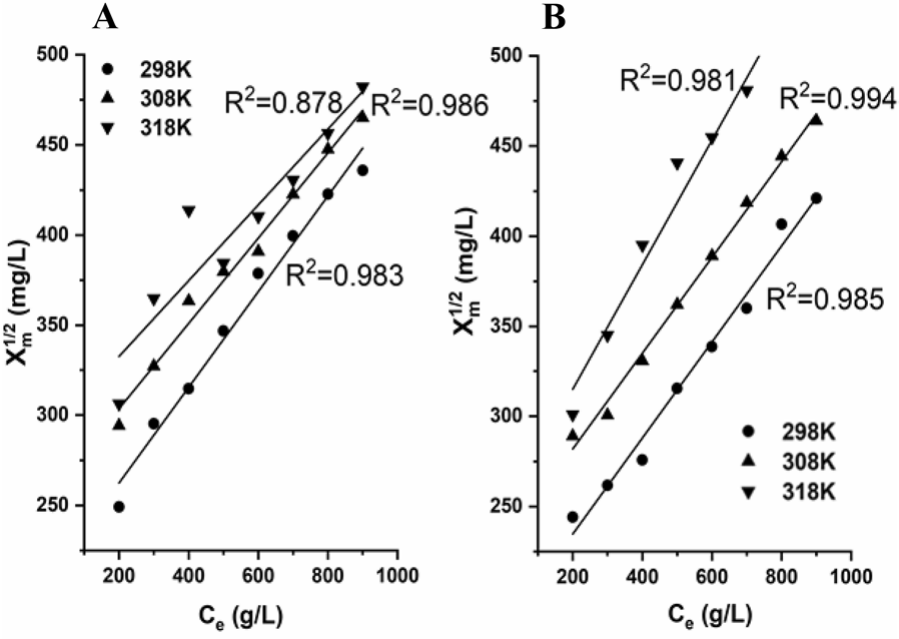
Flocculation model fitting results of OL-PS-DVB (**A**) and MA-PS-DVB (**B**)

At high 1,3-PDO concentration, the sorbent concentration effect was generated in the resin, the resin adsorption decreased with increasing 1,3-PDO concentration. The Flocculation model was used for the sorbent concentration-effect fitting (shown in Fig. 4). This indicates that the form and disintegration between the extractant and 1,3-PDO in the extractive adsorption are similar to the process between flocs and particles in flocculation. The extractive adsorption area in the resin is a function of particle concentration. The article revealed a possible relation between adsorption and flocculation theories which is similar to this result (Helmy et al. 2000).

### Dynamic extractive adsorption

**Fig. 5:**
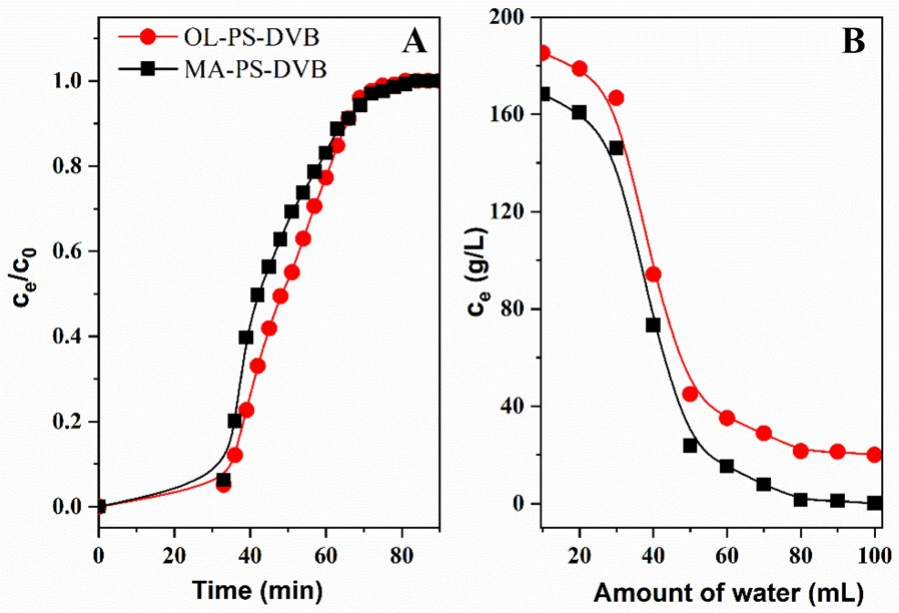
The breakthrough curve of OL-PS-DVB and MA-PS-DVB (**A**); The elution curve for 1,3-propanediol of OL-PS-DVB and MA-PS-DVB (− 20) (**B**)

The breakthrough curve is an important way to describe the adsorption process which reflects the adsorption rate and saturated adsorption capacity of the adsorbent. The dynamic adsorption breakthrough curve for 1,3-PDO was investigated and the result is shown in Fig. 5A. The dynamic adsorption capacity of OL-PS-DVB and MA-PS-DVB was 312 mg/g and 267 mg/g, equivalent to 91.8 mg/mL and 78.4 mg/mL, respectively. The sodium polystyrene sulfonate resin showed a dynamic adsorption capacity of 5.4 mg/mL at a 1,3-PDO concentration of 56.6 g/L. Similarly, the calcium polystyrene sulfonate resin’s dynamic adsorption capacity is 23.6–25.5 mg/mL at a 1,3-PDO concentration of 239.9 g/L (Hilaly and Binder 2002). The comparison shows that the extractive adsorption resins of OL-PS-DVB and MA-PS-DVB have a higher adsorption capacity than polystyrene sulfonate resin.

The Thomas model and Yoon-Nelson model were used to fit the breakthrough curve. The extractive adsorption resins were fitted well with the Thomas model (shown in Table 4) that R^2^ of OL-PS-DVB and MA-PS-DVB are 0.980 and 0.970, respectively. And the theoretical maximum dynamic adsorption capacity of OL-PS-DVB and MA-PS-DVB is 319 mg/g and 280 mg/g which is similar to the experimental value. Yoon-Nelson model is applied to investigate the breakthrough behavior of extractive adsorption resin and the simulation results are shown in Table 5. The plot of ln [C_t_ / (C_o_-C_t_)] versus t (hr) provides a straight line from which the rated velocity constant (K_YN_) and time required for 50% adsorbate breakthrough (τ) can be obtained from the slope and intercept, respectively (Faizal et al. 2014). Compared with the two resins, OL-PS-DVB has a larger rate velocity constant (K_YN_) which indicates that the adsorption process of OL-PS-DVB requires less time.

**Table 4:** Thomas model fitting results of OL-PS-DVB and MA-PS-DVB

Resin type	Equation	Q_t_(mg/g)	R^2^
OL-PS-DVB	ln(c_0_/c_e_− 1) = − 0.15t + 2.87	319	0.980
MA-PS-DVB	ln(c_0_/c_e_− 1) = − 0.12t + 2.05	280	0.970

**Table 5:** Yoon-Nelson model fitting results of OL-PS-DVB and MA-PS-DVB

Resin type	Equation	τ	*R*^2^
OL-PS-DVB	ln(c_e_/(c_0_ − c_e_)) = 0.15t − 7.32	48.80	0.984
MA-PS-DVB	ln(c_e_/(c_0_ − c_e_)) = 0.13t − 5.88	45.23	0.970

For industrial scale-up extraction, one of the major problems is how to reach well-mixed. Bigger reactors increase the difficulty of mass transfer which usually requires a premixer. However, in multistage extraction, extra equipment made the downstream process more intricate. The resin structure could enhance the mixing effect and mass transfer efficiency which could simplify the separation process.

### The elution of the synthesized PS-DVB resins

While the resin was saturated, water was used for elution and the elution curve is shown in Fig. 5B. Combine the eluate of OL-PS-DVB and determine the concentrations of main products in the eluent. The concentrations of 1,3-PDO, butyric acid, acetic acid, and glycerol are 24.7, 5.8, 4.6, and 3.3 g/L, respectively. Calculated by eluent content and adsorption capacity for the OL-PS-DVB, the recovery of 1,3-propanediol, butyric acid, acetic acid, and glycerol were 95.3%, 93.5%, 93.1%, and 88.8%, respectively. Similarly for MA-PS-DVB, the recovery of 1,3-propanediol, butyric acid, acetic acid, and glycerol were 97.1%, 94.7%, 93.3%, and 90.3%, respectively. The protein and salt content in the eluent was determined. However, using resin to separate 1,3-PDO has a dilution effect during the elution process. After excluding the solvent dilution effect, the inorganic salt and protein removal rates were 93.8% and 90.9%, respectively. It shows that extractive adsorption PS-DVB resin can effectively remove impurities and inorganic salts in the fermentation broth.

### Recycle of resin

**Fig. 6:**
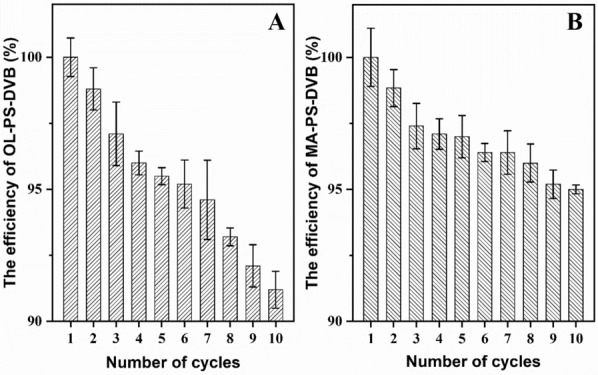
The efficiency of recycled OL-PS-DVB and MA-PS-DVB

The synthesized PS-DVB resins were recycled and reused for 1,3-PDO adsorption and the results are shown in Fig. 6. Due to the hydrophobicity of the extractant, the resin absorption capita does not decrease significantly. After 10 times recycling, OL-PS-DVB still has 89.7% extraction adsorption capacity. Owning to mixed extractant has stronger hydrophobicity, MA-PS-DVB has better circulation performance that 92.4% extraction adsorption capacity still be reached after 10 times recycling. On the whole, extractive absorption OL-PS-DVB and MA-PS-DVB macroporous resins have a good performance in recycling.

## Conclusions

In this study, extractive adsorption was first proposed and investigated for 1,3-PDO separation from fermentation broth. The novel extractive adsorption PS-DVB macroporous resins using n-octanol (OL-PS-DVB) and mixed alcohols as porogen (MA-PS-DVB) were synthesized, respectively. FTIR results indicated that the alcoholic porogen was successfully enveloped in the synthesized resins of OL-PS-DVB and MA-PS-DVB. The hydroxyl group content of OL-PS-DVB and MA-PS-DVB was 7.4% and 6.1%, respectively. Correspondingly, n-octanol and mixed alcohols in OL-PS-DVB and MA-PS-DVB were 56.3% and 56.7%, respectively. According to the BET model, the specific surface areas of OL-PS-DVB and MA-PS-DVB were 48.7 and 17.4 m^2^/g, respectively. In addition, the average pore diameters of OL-PS-DVB and MA-PS-DVB were 19.2 and 21.7 nm, respectively. The static adsorption capacity of OL-PS-DVB and MA-PS-DVB for the concentrated 1,3-PDO broth illustrated that the two novel resins performed better than the control resin (OE-PS-DVB), being 511 and 473 mg/g vs. 102 mg/g, i.e., 5 and 4.6 times, respectively. Calculated by the Langmuir equation, the theoretical static maximum adsorption capacities of MA-PS-DVB could reach 516, 595, and 629 mg/g at 298, 308, and 318 K, respectively. It should be pointed out that the sorbent concentration effect was generated in the two extractive adsorption resins at high 1,3-PDO concentrations. Moreover, this sorbent concentration effect was described well according to the Flocculation model. The dynamic adsorption behavior of OL-PS-DVB and MA-PS-DVB fitted well with Thomas and Yoon-Nelson model. The theoretical maximum dynamic adsorption capacity of OL-PS-DVB and MA-PS-DVB was 319 mg/g and 280 mg/g, respectively. Compared to OL-PS-DVB, MA-PS-DVB showed better performance in the recovery yield of 1,3-PDO and other byproducts, the removal rates of the inorganic salt and protein, and the efficiency of recycled resin. For MA-PS-DVB, the recovery of 1,3-PDO, butyrate acid, acetic acid, and residual glycerol was 97.1%, 94.7%, 93.3%, and 90.3%, respectively. Considering the solvent dilution effect, the inorganic salt and protein removal rates were 93.8% and 90.9%, respectively. The extraction adsorption capacity of 92.4% of MA-PS-DVB still remained after 10 times recycling.

## Data Availability

All data generated or analyzed during this study are included in this article.
